# Knowledge, attitudes, and practices related to soil-transmitted helminth infections among residents of Bata district, Equatorial Guinea; a cross-sectional study

**DOI:** 10.1186/s12889-024-19528-0

**Published:** 2024-07-23

**Authors:** Gertrudis Ribado Meñe, Jean Claude Dejon-Agobé, Basilio Micha Aboho Angue, Maximiliano Fero Meñe, José Manuel Esara Echube, Salim Abdulla, Ayôla Akim Adegnika

**Affiliations:** 1 National University of Equatorial Guinea/Environmental Faculty, Malabo, Equatorial Guinea; 2 Ecole Doctorale Régionale d’Afrique Centrale, Franceville, Gabon; 3 Centre de Recherche Médicales de Lambaréné (CERMEL), Lambaréné, Gabon; 4 Institute of Tropical Medicine, University of Tubingen, Tubingen, Germany; 5 Medical Care Development Global Health, Malabo, Equatorial Guinea; 6 Department of Intervention, Ifakara Health Institute, Dar–es-Salaam, Tanzania; 7 Department of Parasitology, Leiden University Medical Center, Leiden, the Netherlands; 8 German Center for Infection Research, Tubingen, Germany

**Keywords:** Knowledge, Attitude, Practice, Soil-transmitted helminths, Equatorial Guinea

## Abstract

**Background:**

Soil-transmitted helminth (STH) infection control remains a priority in endemic regions where local epidemiological data are needed for sustainable control strategies, particularly regarding population knowledge, attitudes, and practices (KAP). Our work assessed KAP toward STH infection and associated factors among residents of Bata district, Equatorial Guinea.

**Methods:**

A community-based cross-sectional study was conducted among 14 randomly selected communities in the Bata district. Eligible participants were interviewed face-to-face using a standardized questionnaire. Participants aged under 18 years were interviewed in the presence of their parents or legal guardians. For participants aged less than ten, a simplified version of the main questionnaire was developed focusing on children’s practices toward STH and was administered to their parents or legal guardians.

**Results:**

A total of 399 participants were included in the present analysis. Among them, 58% responded to the main questionnaire. The mean (± SD) age of participants aged 10 and over was 37.5 (± 22.2) years, and 60% of them were females, while the mean (± SD) age of those aged less than ten was 5.0 (± 2.5) years. The respondents’ overall knowledge, attitudes, and practices to STH were rated as bad (33%), very good (77%), and good (55%), respectively. Knowledge was significantly associated with education level (*p* = 0.04) with the knowledge level lower for participants with no formal education than for those with secondary/university education (β = -0.56, 95% CI: -1.00 – -0.12, *p* = 0.01); Appropriate attitudes level was significantly associated with occupation (*p* = 0.02) and education levels (*p* = 0.049) with the appropriate attitude level lower for students than for farmers/fishers (β = -1.24, 95% CI: -2.17—-0.32, *p* = 0.01) and for primary-level participants than for those with secondary/university education (β = -0.68, 95% CI: -1.23—-0.13, *p* = 0.02); while appropriate practice level were significantly associated with age (*p* = 0.01), occupation (*p* = 0.01), and education (*p* = 0.02), with the appropriate practices level increasing with age (β = 0.03, 95% CI: 0.005 – 0.05, *p* = 0.01) and lower in participants with no formal education than in those with secondary/university education (β = -1.19, -2.05 – -0.32, *p* = 0.007).

**Conclusion:**

The present study revealed a lack of knowledge about STH in the study population, particularly regarding disease causes and transmission ways, highlighting the need for the implementation of integrated health education strategies, both at the community and school levels.

**Supplementary Information:**

The online version contains supplementary material available at 10.1186/s12889-024-19528-0.

## Background

Among over 20 diseases classified as Neglected Tropical Diseases (NTDs), soil-transmitted helminth (STH) infections are the most prevalent [1]. The main agents responsible for these infections in human populations are *Ascaris lumbricoides*, *Trichuris trichiura,* hookworms (*Ancylostoma duodenal* and *Necator americanus*), and *Strongyloides stercoralis*. Although STH are transmitted mostly in tropical and subtropical regions particularly in areas with poor sanitation, limited access to safe water, and limited economic resources for proper hygiene practices [2]. They constitute a global public health concern. Indeed, there are distributed all over the world, with more than 1.5 billion people infected [3]. In the World Health Organization (WHO) African region, an estimated 350 million individuals are considered at risk for STH infections [4].

STH infections produce a great variety of symptoms that are expressed depending on the parasite infection load. These symptoms include diarrhea, abdominal pain, micronutrient deficiencies, malnutrition, malaise and weakness [5]. Children are the most affected population group, with great impact on their physical growth and cognitive development [6, 7]. In co-endemic areas, STH infections can affect the morbidity of other infectious diseases, such as human immunodeficiency virus [8], malaria [9], and tuberculosis [10]. The morbidity related to STH infection places endemic societies in a state of continuous poverty, making the disease a major public health issue [5, 11].

To attain the WHO 2030 road map for eliminating STHs as a public health problem [12], the WHO proposed periodic community mass deworming for the control of disease morbidity, and the availability of safe water, proper sanitation, hygiene (WASH) and health education for the exposed population to reduce disease transmission [12–14]. Community deworming is the most widely implemented intervention worldwide and is considered fundamental in terms of cost and effectiveness [11, 15]. For the control of disease transmission, providing safe water and adequate sanitation aims at reducing environmental fecal contamination, while implementing health education aims at reducing inappropriate attitudes and practices of the population toward the disease. This integrated approach is highly recommended for successful control programs [5, 11]. In 2020, Nath and collaborators [16] reported a significant improvement in the knowledge of the population on STH infections after health education intervention, as well as in STH preventive behaviors and Mass Drug Administration (MDA) involvement attitudes [16], demonstrating that the integration of health education can be a key element to improve STH control in endemic areas. Therefore, for a tailored health education program, the knowledge, attitude, and practice (KAP) of the population toward STH need to be assessed to, for instance, understand the population knowledge gap and misconception of the disease, the risk-full attitudes and practices and their willingness to participate in the implementation of control strategies. KAP surveys are indeed known as basic tools for guiding the proper development of health education strategies [11, 17].

Equatorial Guinea (EG) is a central African country known to be highly endemic for STH [8, 18–20]. Indeed, the country is reported among the 20 nations with the highest prevalence of STH before 2003, and among the 12 from 2003 to 2018 [20], indicating the necessity to improve the current situation of STH control in the country. In that vein, if school-aged children and peri-urban areas have previously reported to be at greater risk of STH infections in the most populated district of the country [18], the specific contribution of the population is not yet known. As the country has adhered to the WHO’s recommendations for the control of STH [21], the objective of the present study was to assess for the first time in the country the knowledge, attitudes, and practices of the population toward STH infections, which could contribute to the tailored design of a health education strategy and the optimization of other STH control strategies.

## Methods

### Study area

**Fig. 1:**
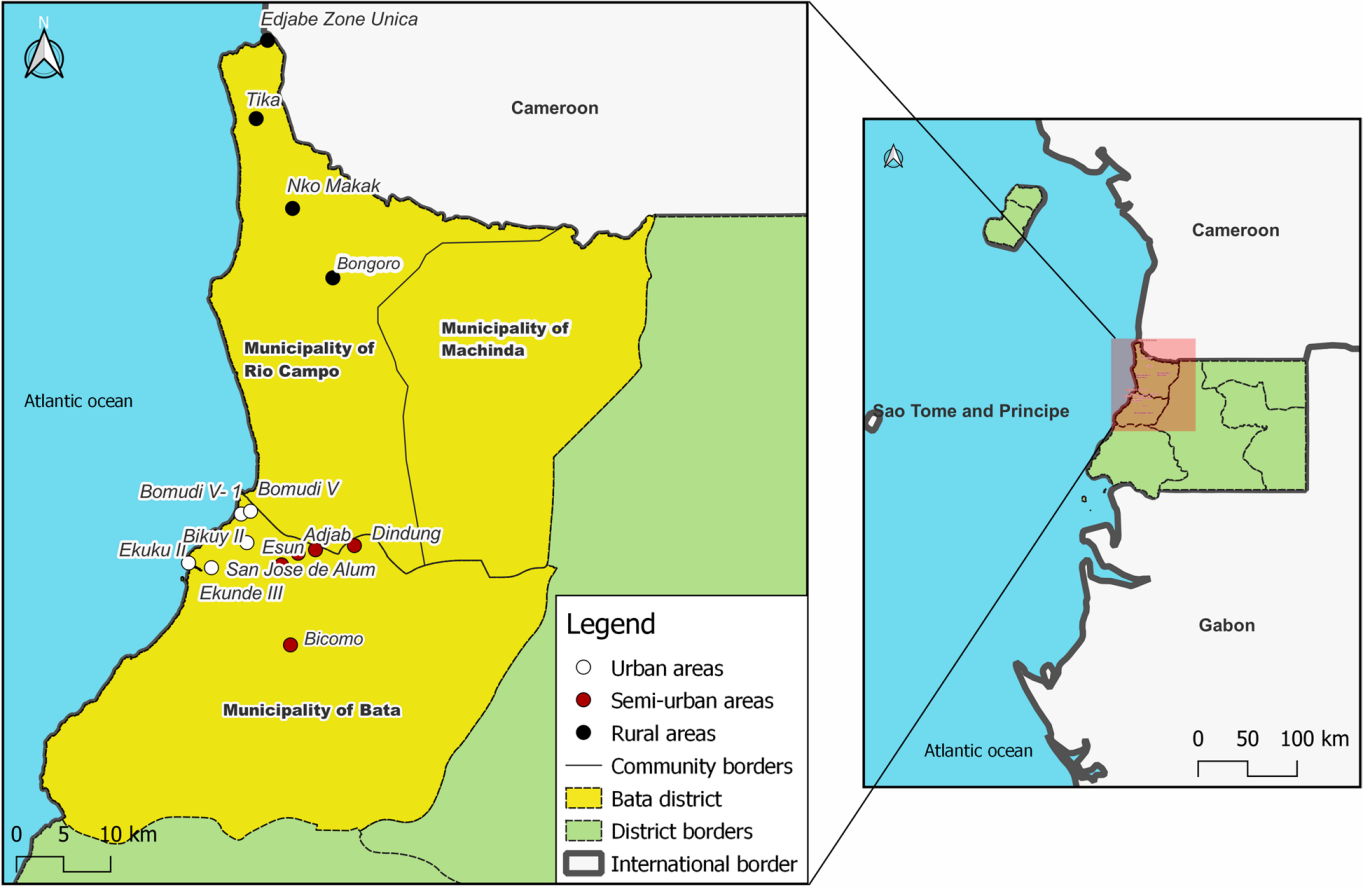
Map showing the geographical location of Equatorial Guinea (Right panel) and communities at Bata district were the study was conducted (Left panel). The base layer for the country and national border shape was obtained from the ^©^2018–2022 GADM; an open license database (https://gadm.org/download_country.html). QGIS version 3.22.16 was used to create the map

The EG, a country located in central Africa, comprises the continental or Rio Muni mainland and the insular Bioko and Annobon islands (Fig. 1). The study was conducted in the district of Bata, one of the 19 districts of the country [22]. Bata city is the capital of Bata district, one of the two main cities in the EG, and it is in the coastal zone of the mainland. It is the largest district in the country, with 25% of the total country population [22]. The survey was conducted in two out of the three municipalities of the Bata district, namely the municipalities of Bata and Rio Campo, where a 60% prevalence of any STH was recently reported [18]. In terms of WASH, 55% of the population have improved water sources; where, 21% are public sources; regarding sanitation; 39.7% have access to improved non-shared facilities, for house floor materials, 54% are cement floors; and 47.4% have a hand washing specific place [23]. The main occupations of the local populations are artisanal fishing, hunting, subsistence agriculture and trading [22].

### Study design and study population

The study was a cross-sectional household community KAP survey carried out from October 2020 to January 2021. Volunteers who lived in the selected communities for at least three months before the survey and who were aged one year or older were eligible to participate in the survey.

### Sample size consideration

The present survey was conducted concomitantly with a study aiming to determine the prevalence of STH infection in EG, particularly the Bata district, from which the initial sample size was calculated [18]. From the total of 403 participants initially included, 399 were considered in the present analysis as they were able to complete the questionnaires. Of them, 233 (58%) were aged 10 years and above. Considering an expected prevalence of 50% (for a maximum possible sample size) and using the formula of sample size calculation for surveys as described by Charan et al. [24], 233 participants give us a precision of 6.4% in the estimation of the proportion of participants’ appropriate knowledge, adequate attitudes or practices regarding STH infection. In addition, the remaining 166 participants were aged less than 10 and therefore specific questions on their practices towards STH were directed to their parents/guardians.

### Sampling and study procedures

The Bata district is divided into three municipalities: Bata, the most populous; Machinda; and Rio Campo. Each municipality is divided into a neighborhood community for the urban zone or into village communities for the peri-urban and rural zones. Participants were selected by applying a three-stage sampling procedure. The first stage consisted of a random selection of two out of the three municipalities (Bata and Rio campo), and the second stage included a probability proportion to the population size in each unit site. In both municipalities, we selected five neighborhoods (representing the urban zone), five peri-urban areas and four villages (representing the rural zone) for a total of 14 communities. The third stage included a convenience selection of households, and where two participants were to be randomly selected and interviewed, based on our eligibility criteria. So, after arriving at a selected household, permission was requested from the person in charge of the household. When permission was granted, the study objectives and participation conditions were explained to the house inhabitants in Spanish and, if needed, in the community local language by a member of the research team originating from the community. Only after that were the volunteers requested to participate. Among those who agreed to participate, eligible volunteers were included in the study after providing informed consent. An identification code was assigned for each participant for confidentiality purposes, and the interviewer administered the questionnaire to the respondent during a face-to-face interview of approximately 15 min. Respondents under 18 years old were interviewed in the presence of their parents or legal guardians. For children aged one to nine years, specific questions on their behavior and practices toward the disease were addressed to their parents or legal guardians.

### Data collection

Participant information was collected through a paper case report form that included the study questionnaire.

### The standardized study questionnaires

The main study questionnaire (see Supplementary material S1) was designed to collect sociodemographic data and to capture the knowledge, attitudes, and practices of the respondents toward STH infection. The questionnaire aimed to capture knowledge about STH from the perspective of the respondents, appropriate and inappropriate respondents’ attitudes, and practices. In the first section of the questionnaire, sociodemographic questions were asked. In the second section of the questionnaire, open- and close-ended questions about respondents’ knowledge were developed. To ensure a better understanding of the respondents, the term “human intestinal parasite” (“worms”) was used instead of “soil-transmitted helminths”. The third and fourth sections of the questionnaire were designed to collect information on the attitudes and practices of the respondents, respectively. For participants’ attitudes, multiple-choice and open-ended questions were included, while for participant practices, only multiple-choice questions were included. The simplified version of the questionnaire (see Supplementary material S2) developed from the main questionnaire was designed to capture sociodemographic, knowledge, attitude and practices of participants’ (aged 10 to 17) related to STH infection. All questionnaires were built in English and translated into Spanish — the national language — pretested, and the final versions were validated by a research committee created for the purpose.

### Statistical analysis

All data collected were digitized using EpiData software version 3.1 and exported to Stata version 13 for analysis. For the respondents’ knowledge assessment, the participants were asked questions with the possibility of providing a maximum of two answers per question. One (1) point was given for each correct answer, while zero (0) points were assigned for each wrong answer, giving a possible total score of six (6) (see Supplementary Table S1). For attitudes and practices assessment, answers to the closed-ended questions were quoted on one (1) mark if correct, while a wrong answer was quoted on zero (0) mark, giving a total possible score of nine (9) and 14, respectively (see Supplementary Table S2 and S3). For knowledge, attitudes, and practices, the mean score for the whole study population was calculated and translated as a percentage. The population level of knowledge, attitudes, and practices was then classified as “bad” if the calculated percentage was less than 50%, “good” if between 50 and 69%, “very good” if between 70 and 89%, and "excellent” if equal to or greater than 90%.

For descriptive statistics, categorical variables were summarized in numbers and proportions, while continuous variables were described as the mean and standard deviation (SD) when normally distributed. The associations between knowledge, attitudes and practices and the sociodemographic characteristics of the respondents were computed using first a simple linear regression model and expressed with the β coefficient and its 95% confidence interval (95% CI). If more than one variable had a *p value* equal to or less than 0.25, adjusted analysis was performed for all variables included in the final model. A *p value* less than 0.05 was considered to indicate statistical significance.

## Results

### Study population characteristics

As presented in Table 1, the mean (± SD) age of the 233 participants (aged from 10 years and above) who responded to the questionnaire was 37.5 (± 22.2) years, with 60% female representation. Most of them (104, 45%) lived in urban areas, while a majority (100, 43%) had a primary education level. Eighty-six (37%) of them were students, and 71 (30%) were farmers/fishermen. For the 166 participants (aged from one year to nine years old) for whom specific questions were directed to their parents or guardians, the mean (± SD) age was 5.0 (± 2.5) years, with 55% male representation. Most of them (72, 43%) lived in urban areas, and more than half (112, 68%) had a primary education, while the remaining 54 (23%) participants had no formal education.

**Table 1:** Sociodemographic characteristics of 399 study participants per age group of inclusion

Sociodemographic characteristics	Participants aged 1 to 9 years old		Participants aged 10 and above	
	**n**	**%**	**n**	**%**
**Total**	166	41.6	233	58.4
**Mean (SD) age**	5.0	(2.5)	37.5	(22.2)
**Sex**				
Females	74	44.6	140	60.1
Male	92	55.4	93	39.9
Female-to-male Sex-ratio	1.2	-	1.5	-
**Locality**				
Urban	72	43.4	104	44.6
Peri—urban	63	37.9	70	30.0
Rural	31	18.7	59	25.3
**Occupation**				
Civil servant	-	-	26	11.2
Farmer/Fisherman	-	-	71	30.5
Student	-	-	86	36.9
Trader	-	-	26	11.2
Unemployed	-	-	24	10.3
**Education**				
No formal education	54	32.5	57	24.5
Primary	112	67.5	100	42.9
Secondary/University	-	-	76	32.6

### Distribution of background knowledge about STH infection among the study population

In Table 2, we present the background information on STH infection knowledge of the 233 participants who responded to the questionnaire. Of them, 184 (79%) respondents had already heard about human intestinal worms, while 191 (82%) of them considered it a disease. The principal places where they heard about STHs for the first time were at home, representing less than half (82, 35%) of the respondents.

**Table 2:** Distribution of the background knowledge of the study population on STH infection

Question on respondent knowledge on STH infection	Study participants	
	**n**	**(%)**
**Have ever heard about human intestinal worms**		
No	49	21.1
Yes	184	78.9
**Consider intestinal worm as a disease**		
No	42	18.0
Yes	191	82.0
**Where did you hear about intestinal worms for the first time?**		
At School	34	14.6
At home	82	35.2
Through television	0	-
At the health center/Hospital	61	26.2
During health campaign	6	2.6
Not answer	50	21.5

**Fig. 2:**
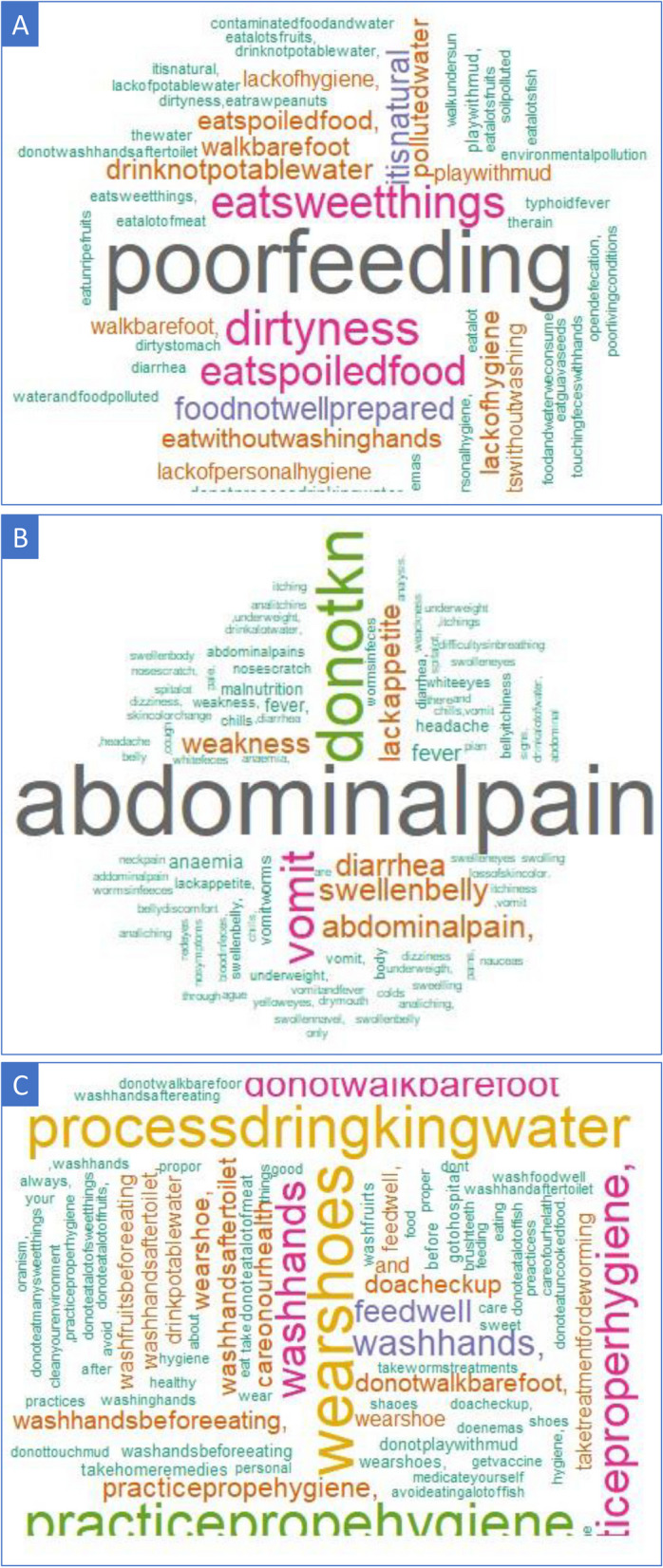
Word cloud map showing the most frequent answers given by the responders to STH infection

The distribution of knowledge on the causes, symptoms, and prevention of STH infection among the 233 participants who answered the questionnaire is summarized in Table 3. On the question about the cause of STH infection, one out of every three respondents (76; 33%) gave correct answers, while approximately one out of two respondents gave correct answers about disease symptoms (140; 60%) and disease prevention (116; 50%). The remaining participants either never heard about the STH or did not know or provided a wrong answer. Figure 2 provides details on the frequency of the answers given by the responders regarding STH infection. For the cause of disease, the most frequent answers considered correct were “poor feeding" “dirtiness”, and “eat food in bad conditions”; while the most wrong answers provided were “Eat sweet things” and “It is something natural” (see Fig. 2A). For disease symptoms, “abdominal pain” and “vomiting” were the main correct symptoms frequently mentioned, while “swelling body and eyes”, followed by “change in skin color”, were the wrong answers most frequently reported by the study population (see Fig. 2B). With respect to knowledge about disease prevention, “practice hygiene”, “wearing shoes”, and “washing hands before eating and after toilet” were the most frequent preventive measures indicated by the study population, while “medical checkup” was the main wrong answer mentioned as a preventive measure for STH. (See Fig. 2C).

**Table 3:** Distribution of the respondents’ knowledge of soil-transmitted helminth (STH) infection causes, symptoms, and prevention

Question on respondent knowledge on STH infection	Study participants	
	**n**	**%**
**Knowledge about the cause of soil-transmitted helminth infection**		
Never heard about STH/Don’t know/Wrong answer	157	67.4
Correct answer provided	76	32.6
**Knowledge about symptoms related to soil-transmitted helminth infection**		
Never heard about STH/Don’t know/Wrong answer	93	39.9
Correct answer provided	140	60.1
**Knowledge about prevention measures for soil-transmitted helminth infections**		
Never heard about STH/Don’t know/Wrong answer	117	50.2
Correct answer provided	116	49.8

### Attitudes of respondents toward STH infection

The results of the participants’ attitudes toward the disease are presented in Table 4. For the question of who is considered the most at-risk population to be infected with STH, 164 (70%) of the respondents indicated “anyone”, while 53 (23%) indicated “children”. The best place to seek solutions in case of STH infection, 213 (91%) of the respondents indicated hospitals, while 7 (3%) and 8 (3%) indicated pharmacy and home remedies, respectively. Among those who mentioned home remedies, a 36-year-old female participant who lived in rural areas argued, “*When I am sick, I must travel to the main city, that means paying for transportation and then for consultation and medicines. Since I do not have enough money to pay for that, I prefer to take home remedies*”. To determine whether pharmaceutical drugs or home remedies are the best, 173 (74%) of the respondents considered pharmaceutical drugs the best, while 16 (7%) considered both or home remedies the best. Assessing the acceptance of drug donation as a community mass drug administration, 129 (55%) respondents indicated “Yes”, while 27 (12%) indicated “No” for diverse reasons. In this regard, a 46-year-old male residing in an urban area stated, “*We don´t have to take medicine without medical prescriptions*”, while a 51-year-old female residing in a rural area stated, “*I will not accept the donation because I’m not sick*”.

**Table 4:** Distribution of answers to questions about attitudes toward STH infection among the 233 study participants aged ten and over

Respondents’ attitudes on Soil-transmitted Helminths infections	Study population	
	**n**	**%**
**Who do you consider as most at-risk to be infected with intestinal worms?**		
Anyone	164	70.4
Children	53	22.7
Women	3	1.3
The poor	5	2.2
I don’t know	8	3.4
**When a member of your family has intestinal worms, where would you go for solution?**		
At the hospital	213	91.4
To the pharmacy	7	3.0
I will use home remedy	8	3.4
I can’t tell	5	2.2
**Which treatment do you consider as better; pharmaceutical drugs or home remedies?**		
Pharmaceuticals drugs	173	74.2
Both	16	6.9
Home remedies	17	7.3
I can’t tell	13	5.6
I don’t know	14	6.0
**Would you accept a donation of treatment if it is offered in your community?**		
No	27	11.6
Yes	129	55.4
I don’t know	77	33.0

### Practices of respondents regarding STH infection

Table 5 presents the results from the 233 respondents’ practices regarding STH infections. Concerning the question on how often study participants wash their hands with soap; 59 (25%) stated to always do so before cooking, 70 (30%) before eating, and 70 (30%) always do so after eating. Regarding the question of how often they wash fruits and vegetables before eating them, 75 (32%) participants said always, while 11 (5%) never did so. On the practice of walking barefoot, 55 (24%) participants reported never, while 23 (10%) reported always walking barefoot. More than half of the respondents (147; 63%) never treated tap water before drinking. For open defecation, 144 (62%) respondents stated that they sometimes defecated in open places, while 21 (9%) always defecate in open places.

**Table 5:** Respondents’ practices toward soil-transmitted helminth infections

Respondent Practices	Participants aged 1 to 9 years old (*N* = 166)		Participants aged 10 years and over (*N* = 233)	
	**n**	**%**	**n**	**%**
**Do you wash your hands with soap before cooking?**				
Always	-	-	59	25.3
Sometimes	-	-	157	67.4
Never	-	-	17	7.3
**Do you wash your hands with soap before eating?**				
Always	17	10.2	70	30.0
Sometimes	131	79.0	151	64.8
Never	18	10.8	12	5.2
**Do you wash your hands with soap after toilet?**				
Always	18	10.8	70	30.0
Sometimes	121	72.9	143	61.4
Never	27	16.3	20	8.6
**Do you walk bare foot?**				
Always	44	26.5	23	9.9
Sometimes	111	66.9	155	66.5
Never	11	6.6	55	23.6
**Do you treat tap water before drinking?**				
Always	-	-	23	9.9
Sometimes	-	-	63	27.0
Never	-	-	147	63.1
**Do you defecate in open places?**				
Always	27	16.3	21	9.0
Sometimes	89	53.6	144	61.8
Never	50	30.1	68	29.2
**Do you wash fruits and vegetables well before eating?**				
Always	-	-	75	32.2
Sometimes	-	-	147	63.1
Never	-	-	11	4.7
**Do your child bites his/her nails**				
Always	20	12.0	-	-
Sometimes	71	42.8	-	-
Never	75	45.2	-	-
**Does your child play with mud**				
Always	63	38.0	-	-
Sometimes	86	51.8	-	-
Never	17	10.2	-	-

The practices of 166 participants aged one to nine years toward STH infection were investigated. To determine whether they washed their hands with soap before eating or after using the toilet, parents indicated never for 18 (11%) and 20 (16%) of the children, respectively, and sometimes for 131 (79%) and 121 (73%), respectively. For 111 (67%) and 44 (26%) children, parents indicated that they sometimes or always walked barefoot, respectively. For the question on open defecation, 89 (54%) and 50 (30%) parents indicated that they sometimes and never defecated in open places, respectively. To determine whether their child bites his/her nails, 71 (43%) parents indicated sometimes, while 20 (12%) indicated always. Regarding play habits, 86 (52%) and 63 (38%) parents of children indicated that their child sometimes or always played with mud, respectively.

### Factors associated with STH knowledge, attitudes, and practices

As presented in Table 6, the mean score for knowledge was 1.99 (33%) out of a possible total of 6, classifying the knowledge level of the population as “bad”. According to the univariate analysis (Table 7), only education was associated with knowledge level (*p* = 0.04). Compared to participants with a secondary or university education, those with no formal education had a significantly lower knowledge level (β = -0.56, 95% CI: -1.00 – -0.12, *p* = 0.01), while no difference was observed for those with a primary school education (β = -0.32, 95% CI: -0.71 – 0.06, *p* = 0.10).

**Table 6:** Overall knowledge, attitudes, and practices scores and their interpretations

	**Overall score**		
	**Mean/Total**	**%**	**Interpretation**
Knowledge	1.99/6	33.2	Bad
Appropriate attitudes	6.97/9	77.4	Very good
Appropriate practices	7.72/14	55.1	Good

**Table 7:** Univariate linear regression assessing associations between participants correct knowledge, appropriate attitudes, and practices toward soil-transmitted helminths and sociodemographic characteristics

	**Knowledge**			**Appropriate attitudes**			**Appropriate practices**		
	β	95%CI(β)	*p value*	β	95%CI(β)	*p value*	β	95%CI(β)	*p value*
**Age**	**-0.001**	**1.71 – 2.36**	**0.73**	**0.02**	**0.01 – 0.03**	**0.001**	**0.02**	**0.01 – 0.03**	**0.006**
**Sex**			**0.36**			**0.67**			**0.44**
Female	ref			ref			ref		
Male	-0.16	-0.50 – 0.18	0.36	-0.10	-0.56 – 0.36	0.67	-0.24	-0.85 – 0.37	0.44
**Locality**			**0.31**			**0.40**			**0.048**
Urban	ref			ref			ref		
Peri urban	-0.30	-0.69 – 0.10	0.14	0.13	-0.38 – 0.66	0.61	-0.80	-1.50 – -0.11	0.02
Rural	-0.19	-0.61 – 0.22	0.36	0.38	-0.18 – 0.94	0.18	0.02	-0.71 – 0.76	0.95
**Occupation**			**0.41**			** < 0.001**			**0.002**
Farmer/Fisherman	ref			ref			ref		
Civil servant	0.23	-0.35 – 0.81	0.44	0.48	-0.27 – 1.22	0.21	1.35	0.34 – 2.35	0.008
Student	0.20	-0.39 – 0.43	0.92	-0.98	-1.50 – -0.45	< 0.001	-0.27	-0.10 – 0.43	0.44
Trader	0.53	-0.48 – 1.12	0.07	0.52	- 0.23 – 1.26	0.17	1.61	0.61 – 2.62	0.001
Unemployed	0.71	-0.53 – 0.67	0.81	0.12	- 0.65 – 0.90	0.75	0.70	-0.33 – 1.73	0.18
**Education**			**0.04**			** < 0.001**			**0.03**
Secondary/University	ref			ref			ref		
Primary	-0.32	-0.71 – 0.06	0.10	-0.96	-1.45 – -0.45	< 0.001	-0.90	-1.59 – -0.22	0.01
No formal education	-0.56	-1.00 – -0.12	0.01	0.02	-0.56 – 0.60	0.94	-0.77	-1.56 – 0.01	0.05

Regarding attitudes toward STH infections, the mean score for appropriate attitudes was 6.97 (77%) out of a possible total of 9, classifying the population-appropriate attitudes toward STH infection as “very good” (Table 6). According to the bivariate analysis (Table 7), occupation (*p* < 0.001) and education (*p* < 0.001) were significantly associated with attitudes. After adjusting for occupation and education, an association remained between appropriate attitudes and both variables. Compared to farmers/fishers, students had a lower level of appropriate attitudes (β = -1.24, 95% CI: -2.17 – -0.32, *p* = 0.009), while no difference was observed with the other modalities of occupation: civil servant (*p* = 0.86), trader (*p* = 0.46), and unemployed (*p* = 0.83). For education, a significantly lower appropriate attitude level was observed among participants with a primary education level than among those with a secondary/university education level (β = -0.68, 95% CI: -1.23—-0.13, *p* = 0.02), while no difference was observed among participants with no formal education (*p* = 0.31).

Concerning the practices of the study population toward STH infections, the mean score for appropriate practices was 7.72 (55%) out of a possible total of 14, classifying the practices level of the population as “good” (Table 6). According to the univariate analysis, as presented in Table 7, age (*p* = 0.006), locality (*p* = 0.048), occupation (*p* = 0.002), and education (*p* = 0.03) were associated with appropriate practices. After adjustment for those variables (Table 8), associations remained for age (*p* = 0.01), occupation (*p* = 0.01), and education (*p* = 0.02). With respect to age, the appropriate practices increase by 0.03 when age increases by one (β = 0.03, 95% CI: 0.005 – 0.05, *p value* = 0.01). For occupation and compared to farmer/fisherman, traders had a statistically significant lower appropriate practices level (β = 1.81, 95% CI: 0.76 – 2.87*, p* = 0.001), while no difference was observed for civil servants (*p* = 0.11), students (*p* = 0.69), or unemployed individuals (*p* = 0.50). Compared to participants with a secondary/university education, those with no formal education had a statistically significant lower level of appropriate practices (β = -1.19, 95% CI: -2.05 – -0.32, *p* = 0.007), while no difference was observed for participants with a primary education level (*p* = 0.35).

**Table 8:** Multivariate analysis of the associations between appropriate attitudes and practices and sociodemographic characteristics

	**Appropriate attitudes**			**Appropriate practices**		
	β	95%CI(β)	*P value*	β	95%CI(β)	*P value*
**Age**	**-0.007**	**-0.02 – 0.01**	**0.45**	**0.03**	**0.005 – 0.05**	**0.01**
**Locality**			**-**			**0.08**
Urban	-			Ref		
Peri urban	-	-	-	-0.80	-1.54 – -0.06	0.03
Rural	-	-	-	0.19	-0.96 – 0.58	0.63
**Occupation**			**0.02**			**0.01**
Farmer/Fisherman	ref			Ref		
Civil servant	0.02	-0.82 – 1.22	0.86	0.92	-0.22 – 2.06	0.11
Student	-1.24	-2.17 – -0.32	0.01	0.25	-1.01 – 1.51	0.69
Trader	0.29	-0.49 – 1.08	0.46	1.81	0.76 – 2.87	0.001
Unemployed	-0.08	-0.86 – 0.70	0.83	0.37	-0.71 – 1.44	0.50
**Education**			**0.049**			**0.02**
Secondary/University	Ref			Ref		
Primary	-0.68	-1.23 – -0.13	0.02	-0.35	-1.08 – 0.38	0.35
No formal education	-0.34	-0.99 – 0.31	0.31	-1.19	-2.05 – -0.32	0.007

## Discussion

The objective of the present survey was to assess the knowledge, attitudes, and practices of inhabitants of Bata districts toward STH infections, which could be helpful for designing health education strategies and optimizing other STH control strategies. Basically, our results indicate a poor level of knowledge but, in contrast, a very good level of appropriate attitudes and good level of appropriate practices of the Bata district population toward STH infections.

Approximately eight out of ten respondents reported having heard about intestinal worm infections, which could indicate that the population is aware of the disease. A similar high proportion of the population aware of the disease was also reported by Oyebamiji and collaborators in southwestern Nigeria with 63% of the study population having heard about intestinal worm infection [25].

In the present survey, the main source of information on STHs was at home for approximately half of the responders, assuming that information was obtained from friends and relatives via word-to-mouth. Our results are different from those reported by a study conducted in Bangladesh, where the main sources of information were schoolteachers and health workers, although in the context of the absence of a health education program [26]. Our findings could explain the poor overall knowledge of STH infections reported by our community. We can therefore assume that our results highlight a lack of health education programs at the study site and probably in the country, which is mainly needed to inform the communities about STH risk factors. Indeed, being informed on the risk factor of STH infection could help the population to apply adequate hygienic habits in order to reduce the risk of infection.

Even though the knowledge level on the causes of STH infections was particularly poor in our study population, we found a good level of knowledge on disease symptoms and prevention, probably because the population experienced these two aspects of the disease. The study site is indeed known to be highly endemic for STH infections [18]. Our results differ from those reported in Western Côte d`Ivoire, where respondents possessed good knowledge of STH causes, transmission and prevention measures but as a consequence of community-based interventions, which can explain the difference observed [27].

Respondents’ knowledge of STHs was associated with education, with participants who did not attend school having a lower knowledge level of STH infections than those who attended at least secondary education. Our results corroborate those reported by Nasr et al. [26] where education level was the most important factor associated with respondents’ knowledge. As we are not aware of health education at schools, we can assume that those with secondary or higher education received more information on proper hygiene in general and probably on general disease prevention measures or are more likely to obtain information themselves through reading for example which can therefore explain the gap in STH knowledge between them and the other population. This could thus highlight the need for the integration of schools and community-based health education strategies in our study areas for the spread of wider prevention information. Indeed, a positive impact of health education and health promotion measures on STH infections in endemic areas has been reported [28].

The study population reported an appropriate attitude toward STH infections. A similar result was reported in Thailand by Narkkul et al. [29] in 2022 but in village health volunteers, where the authors found that a good attitude was associated with training in parasitic control measures for those volunteers [29]. On the one hand, we hypothesize that the good level of appropriate attitudes in our study population is not rooted in STH infection control strategies, as such programs are not implemented in the country, but rather in the general attitude of the population toward adequate hygiene. Indeed, to assess the attitudes of our study population toward STH infections, the questionnaire administered to the responders can also be valid for other infections such as human immunodeficiency virus (HIV), tuberculosis (TB), and malaria, although it was clearly about STH infections.

We found occupation and educational level to be factors associated with respondents’ appropriate attitudes. Compared with other occupations and education levels, primary education level was found to be negatively associated with attitude level. Our result is consistent with that reported by Ba and collaborators among students in Nigeria, where respondent attitudes were more positive for students at high levels than for those at lower levels [30]. Indeed, primary school pupils, particularly school-age children, are classified by the WHO as the most at-risk population for STH infections due to less care and less proper hygiene practices, outdoor play habits, and a lack of WASH at schools and at home [11]. Although we reported a good level of attitude in our population, children and students could therefore be the weakest links for the control of STHs in the country. Regarding the population’s attitudes toward STH infection, we call for a specific emphasis on school-based health education strategies at the early school and primary school levels.

A good level of appropriate practices toward STH infections in our population were found. Indeed, we noticed that practices that favor STH transmission, such as walking barefoot, defecating in open places, and particularly for children, playing with mud, are sometimes or never practiced. Similarly, protective factors, such as washing hands with soap before eating and washing fruits and vegetables before consumption, are mostly or always practiced by the study population. As we hypothesized for the attitudes of our population toward STH infection, a good level of appropriate practices observed in the population could not be specifically rooted in STH infection prevention but rather a general way of life, suggesting that the practices of our population do not play a major role in STH transmission in the country. A recent study conducted in Bata district reported for instance that 96% of the population lacked sewage systems connected to their houses [18]. We can assume that environmental factors could mainly explain the high prevalence of STH infections observed in the country. Tropical climate, sandy soils as it is the case for Bata district, local temperatures, in addition to low proportion of sewage systems and the habit of not processing tap drinking water have been found to increase the risk of STH [22, 23, 31–33]. This, however, needs to be properly investigated. We found that age and education level were associated with respondents’ practices. Indeed, a positive association was observed with age and education level, indicating an increase in appropriate practices with increasing age and education level. These results are in agreement with those reported by Le and collaborators in rural areas of Indonesia, where behavior scores presented a positive association with the age and education level of respondents [28]. This result can be explained by adults being more conscious of their hygiene than children are, and people with a high education level are more likely to be in contact with hygienic information and prevention measures. These results could indicate a need to start health education early in life and at low education levels so that children can be aware of appropriate hygiene practices early in life.

Our survey was based on a structured questionnaire with close-ended questions, especially about respondents’ attitudes and practices. We did not include direct observation of the study population. Our finding may therefore be slightly out of keeping with reality. Indeed, in a KAP survey, the inclusion of observation methods is suggested to be able to capture the real reasons behind respondents’ behaviors [34, 35]. Furthermore, our study was conducted in only one out of 19 districts of the whole country, although it is the most populated district, with approximately 25% of the country population. As we must remain cautious about extrapolating our results to the whole country, we think that additional research should be conducted in other districts of the country to obtain a wider understanding of the whole country. Despite the limitations highlighted, the data on our KAP offer relevant information about our population KAP, which could be used for the orientation of control programs focused on population gap knowledge and full risk perception and practices found during the survey.

## Conclusions

Despite the good level of appropriate attitudes and practices of the population toward STH we found in our community, the low knowledge level highlights the need for the implementation of integrated health education strategies at both the school and community levels, with great attention given to the early-age stage and primary school children. Such a strategy could contribute to maximizing the effects of any potential control program for STH infection implemented in the country, which is known to be highly endemic for STH infections.

### Supplementary Information

Supplementary Material 1: Supplementary Table S1. Respondents’ knowledge score calculation. Supplementary Table S2. The respondents’ appropriate attitudes score calculation. Supplementary Table S3. Respondents’ appropriate practices score calculation.

Supplementary Material 2: Supplementary material S1. Study main questionnaire for adults

Supplementary Material 3: Supplementary material S2. Simplify version of the study main questionnaire for children aged 10 to 17 years

## Data Availability

Data sets generated during the current study are available from the corresponding author on reasonable request.

## Abbreviations

CI: Confidence Interval
EG: Equatorial Guinea
HIV: Human Immunodeficiency Virus
KAP: Knowledge, attitudes, and practices
MDA: Mass Drug Administration
NTD: Neglected Tropical Disease
SI: Supplementary information
SD: Standard deviation
STH: Soil-Transmitted helminth
TB: Tuberculosis
WHO: World Health Organization
WASH: Water Sanitation and Hygiene
