# Supplementary material for: Knowledge, attitudes, and practices related to soil-transmitted helminth infections among residents of Bata district, Equatorial Guinea; a cross-sectional study
Source: BMC Public Health. 2024 Jul 23;24:1962. doi: 10.1186/s12889-024-19528-0 (PMC11264363; doi:10.1186/s12889-024-19528-0)
Supplement: Supplementary file 1 — Supplementary Material 1: Supplementary Table S1. Respondents’ knowledge score calculation. Supplementary Table S2. The respondents’ appropriate attitudes score calculation. Supplementary Table S3. Respondents’ appropriate practices score calculation. [file 12889_2024_19528_MOESM1_ESM.docx]

**Supplementary Table S1.** Respondents’ knowledge score calculation

| ***Answers given to questions on knowledge*** | | ***Score*** |
| --- | --- | --- |
| ***Knowledge about the cause of soil-transmitted helminth infection*** | | |
|  | Don’t know | 0 |
|  | Eat sweet things | 0 |
|  | It is something natural | 0 |
|  | Eat many fruits | 0 |
|  | Each many fish/bushmeat | 0 |
|  | The rain | 0 |
|  | Eat food in bad conditions | 1 |
|  | Poor feeding | 1 |
|  | Unsaved water | 1 |
|  | Dirtiness | 1 |
|  | Lack of hygiene | 1 |
|  | Food not well prepared | 1 |
|  | Walk barefoot | 1 |
|  | Drink unsaved water | 1 |
|  | Eating without washing hands | 1 |
|  | Defecate in open places | 1 |
|  | Play with mud | 1 |
| Maximum subtotal score | | 2* |
| ***Knowledge about symptoms related to soil-transmitted helminth infection*** | | |
|  | Don’t know | 0 |
|  | Swelling body and eyes | 0 |
|  | Change of skin color | 0 |
|  | Nose scratching | 0 |
|  | Fever | 0 |
|  | Drinking a lot of water | 0 |
|  | No sign or symptoms | 0 |
|  | Abdominal pain | 1 |
|  | Lack of appetite | 1 |
|  | Under weight | 1 |
|  | Vomits | 1 |
|  | Weakness | 1 |
|  | Diarrhea | 1 |
|  | Swelling belly | 1 |
|  | Anaemia | 1 |
|  | Fever, Diarrhea | 1 |
|  | Swelling belly | 1 |
|  | Vomiting | 1 |
|  | Underweight, Change of skin color | 1 |
|  | Swelling belly | 1 |
|  | Weakness, uncomfortable | 1 |
|  | Presence of worm in the feces | 1 |
| Maximum subtotal score | | 2* |
| ***Knowledge about prevention measures for soil-transmitted helminth infections*** | | |
|  | Don’t know | 0 |
|  | Medical check-up | 0 |
|  | Avoid eating a lot of fish/meet | 0 |
|  | Do not eat unripe fruits and uncooked food. | 0 |
|  | Avoid sweet things | 0 |
|  | Drink home remedies | 0 |
|  | Nothing can be done | 0 |
|  | Don’t play with mud | 1 |
|  | Feed well | 1 |
|  | Practices Hygiene | 1 |
|  | Process drinking water | 1 |
|  | Take preventive treatments | 1 |
|  | Wash hands before eating | 1 |
|  | Wash hands after toilet | 1 |
|  | Wash fruits | 1 |
|  | Wear shoes | 1 |
| Maximum subtotal score | | 2* |
| Maximum total score | | 6 |

*The responder was allowed to give a maximum of two answer

**Supplementary Table S2.** Respondants’ appropriate attitudes score calculation

| ***Possible answer to questions on attitudes*** | | ***Score*** |
| --- | --- | --- |
| **Who do you consider as most at-risk to be infected with intestinal worms** | | |
|  | Children | 3 |
|  | Women | 2 |
|  | The poor | 2 |
|  | Anyone | 1 |
|  | I don’t know | 0 |
| ***When a member of your family has intestinal worms, where would you go for solution?*** | | |
|  | At the hospital | 2 |
|  | To the pharmacy | 1 |
|  | I will use home remedy | 0 |
|  | I can’t tell | 0 |
| ***Which treatment do you consider as better; pharmaceutical drug or home remedies*** | | |
|  | Pharmaceuticals | 2 |
|  | Both | 1 |
|  | Home remedies | 0 |
|  | I can’t tell | 0 |
|  | I don’t know | 0 |
| **Would you accept a donation of treatment if it is offered in your community?** | | |
|  | Yes | 2 |
|  | No | 0 |
|  | I don’t know | 0 |
| Maximum total score | | 9 |

**Supplementary Table S3.** Respondents’ appropriate practices score calculation

| ***Possible answer to question on practices*** | | ***Score*** |
| --- | --- | --- |
| ***Do you wash your hands with soap before cooking?*** | | |
|  | Always | 2 |
|  | Sometimes | 1 |
|  | Never | 0 |
| ***Do you treat tap water before drinking?*** | | |
|  | Always | 2 |
|  | Sometimes | 1 |
|  | Never | 0 |
| ***Do you wash fruits and vegetables well before eating?*** | | |
|  | Always | 2 |
|  | Sometimes | 1 |
|  | Never | 0 |
| ***Do you wash your hands with soap before eating?*** | | |
|  | Always | 2 |
|  | Sometimes | 1 |
|  | Never | 0 |
| ***Do you wash your hands with soap after toilet?*** | | |
|  | Always | 2 |
|  | Sometimes | 1 |
|  | Never | 0 |
| ***Do you walk bare foot?*** | | |
|  | Never | 2 |
|  | Sometimes | 1 |
|  | Always | 0 |
| ***Do you defecate in open places?*** | | |
|  | Never | 2 |
|  | Sometimes | 1 |
|  | Always | 0 |
| Maximum total score | | 14 |
