# Supplementary material for: Knowledge, attitudes, and practices related to soil-transmitted helminth infections among residents of Bata district, Equatorial Guinea; a cross-sectional study
Source: BMC Public Health. 2024 Jul 23;24:1962. doi: 10.1186/s12889-024-19528-0 (PMC11264363; doi:10.1186/s12889-024-19528-0)
Supplement: Supplementary file 2 — Supplementary Material 2: Supplementary material S1. Study main questionnaire for adults [file 12889_2024_19528_MOESM2_ESM.pdf]

Participant ID Number: Co-STH |_ _|_ _|__|__| Participant Initials |__|__| __|

PARTICIPANT DATA COLLECTION FORM (ADULTS)

PROTOCOL: [*V*].1 Dec.06/2020

[CONTROL OF *SOIL TRANSMITTED HELMINTHS IN EQUATORIAL GUINEA]*

Date of the survey:

Date		Month			Year	

Data collection form 1/4

Q1. PARTICIPANT IDENTIFICATION AND SOCIO-DEMOGRAPHICAL INFORMATION		
**Demography**		
Q1.1.- Sex	1. ☐ Male<br>2. ☐ Female	
Q1.2.- Date of birth	|__|__| |__|__|__|__|__|__|__| Day - month - year	Estimated age: |__|__| year old
Q1.3.- What is the name of your community?	……………………………………………………………………	
Q1.4.- How long have you been living here?	|__|__| years	
**Socioeconomic**		
Q1.5.- Which is your education level?	1. ☐No education<br>2. ☐ Primary<br>3. ☐ Secondary<br>4. ☐University<br>5. Other………………………………………………………	
Q1.6.- Which is your principal occupation	1. ☐ Farmer<br>2. ☐ Fisherman<br>3. ☐ Trader<br>4. ☐ Civil servant<br>5. ☐ Student<br>6. ☐ Unemployed<br>7. ☐ Others_______________________________	
**Living conditions**		
Q1.7.- Type of house material	1. ☐ Cement<br>2. ☐ Plank<br>3. ☐ Other	
Q1.8.-Type of house floor	1. ☐ Cemented<br>2. ☐ un-cemented	
Q1.9.- Do you use toilet?	1. ☐ yes => go to question Q1. 11<br>2. ☐ No	
Q1.10.- specify the defecation site	1. ☐ Behind house<br>2. ☐ Open places<br>3. ☐ Bush<br>4. ☐Others…………………………………………………	
Q1.11.- What type of toilet do you use?	1. ☐ Private/ Modern<br>2. ☐ Communal latrine<br>3. ☐ Pit latrine	
Q1.12.- Type of toilet Floor	1. ☐ Cemented<br>2. ☐ Un- cemented	
Q1.13.-Which is your principal drinking water sources	1. ☐ Tap inside house<br>2. ☐ Tap out site house	

	3. ☐ Well<br>4. ☐ Stream<br>5. ☐ Others
**Environmental sanitation**	
Q1.14.- Is it possible to have indirect contamination of your Domestic water?	1. ☐ yes<br>2. ☐ No
Q1.15.- Do you have water Sources contaminated with feaces close to the house?	1. ☐ yes<br>2. ☐ No
Q1.16.- Do you have a sewage system connected to your house?	1. ☐ yes<br>2. ☐ No
Q.1.17.- If No, where do you drain the waste water? (toilet, kitchen)	1. ☐ River<br>2. ☐ Behind house<br>3. ☐ Underground<br>4. ☐ Others………………………………………………….

Data collection form 2/4

Q2.- ASSESMENT OF KNOWLEDGE TOWARDS SOIL-TRANSMITTED HELMINTH INFECTION	
**Knowledge about Soil-transmitted helminth infections**	
Q2.1.- Have you ever heard about human Intestinal worms?	1. ☐ yes<br>2. ☐ No
Q2. 2.- Where did you hear about intestinal worm for the first time?	1. ☐ At school<br>2. ☐ At home (from mouth)<br>3. ☐ At the health care center/Hospital<br>3. ☐ During health campaign<br>4. ☐ Not answer
Q2.3.- Do you considered intestinal worm as a disease?	1. ☐ yes<br>2. ☐ No
Q2.4.-What do you think are the cause of human intestinal worms	1 …………………………………………………………………….<br>2 …………………………………………………………………….<br>3 …………………………………………………………………….
Q2.5.- Do you know the local name of SHT worm?	1. ☐ yes ……………………………………………………………<br>2. ☐ No
Q2.6.- According to you, which are the symptoms that show you that some has Human intestinal worms	1 ……………………………………………………………………<br>2 ……………………………………………………………………<br>3 ……………………………………………………………………<br>4 ☐ I don`t know
Q2.7.-Do you know how any can contact STH	1 ……………………………………………………………………<br>2 ……………………………………………………………………<br>3 ……………………………………………………………………<br>4 ☐ I don`t know
Q2.8.- Which of the following Symptoms are related with the Human Intestinal worms	1. ☐ Abdominal pain<br>2. ☐ Anemia<br>3. ☐ Underweight- and malnutrition<br>4. ☐ Lack of concentration at school<br>5. ☐ Stunted growth<br>6. ☐ Lack of appetite<br>7. ☐ I don't know<br>8. ☐ Others ………………………………………………………
**Knowledge about the transmission of Soil-transmitted helminth infection**	
Q2.9.-What are the social factors that makes us to be infected?	1. ☐ Lack of personal hygiene<br>2. ☐ Lack of information or health education<br>3. ☐ Deficient water supplies.<br>4. ☐ Lack of toilet<br>5. ☐ All above<br>6. ☐ I don't Know<br>7. ☐ Others____

Q2.10.- What are de human factors that makes us to be infected	1. ☐ Walk barefoot<br>2. ☐ Eat without washing hands<br>3. ☐ Eat fruits and vegetable not well washed.<br>4. ☐ Not process water before drinking<br>5. ☐ Not wash hands before cooking<br>6. ☐ Don't wash hands after toilet<br>7. ☐ All above<br>8. ☐ I don`t know<br>9. ☐ Others ________________________________
**Knowledge about the Disease prevention**	
Q2.11.- How can we prevent human intestinal worm infections?	1 ...………………………………………………………………<br>2 ………...…………………………………………………………<br>3 ……...……………………………………………………………<br>4 …...………………………………………………………………<br>5 ...…………………………………………………………………

Data collection form 3/4

Q3.- ASSESMENT OF VOLUNTREE ATTITUDE TOWARDS THE DISEASE.	
**Disease perception**	
Q3.1.- Who do you consider as most at-risk to be infected with intestinal worms?	1. ☐ Anyone<br>2. ☐ Children<br>3. ☐ Women<br>4. ☐ The poor<br>5. ☐ I don't know<br>6. ☐ Others____
Q3.2.- When you or a member of your family has intestinal worms, where would you go for solution?	1. ☐ At the hospital<br>2. ☐ To the pharmacy (self-medication)<br>3. ☐ I will use home remedies<br>4. ☐ I can't tell<br>5. ☐ Others____
Q.3.3.- Which treatment do you consider as better; pharmaceutical drugs or traditional remedies?	1. ☐ Pharmaceuticals drugs<br>2. ☐ Both<br>3. ☐ Traditional remedies<br>4. ☐ I don't know
Q3.4.- Would you accept a donation of treatment if it is offered in your community?	1. ☐ yes<br>2. ☐ No<br>Comment your answer…………………………………………………
Q.3.5.- Would you accept to be treated if you be found positive in helminths	1. ☐ yes<br>2. ☐ No<br>Comment your answer…………………………………………………
Q.3.6.- Would you take the treatment if offered as community program even if you are not tested for helminths	1. ☐ yes<br>2. ☐ No<br>Comment your answer…………………………………………………

Data collection form 4/4

Q4.- ASSESMENT OF VOLUNTEER PRACTICES TOWARDS THE DISEASE.	
**Practices related to exposition**	
Q4.1.- Do you wash your hands with soap before cooking	1. ☐ Always<br>2. ☐ Sometimes<br>3. ☐ Never
Q4.2.- Do you wash your hands with soap before eating?	1. ☐ Always<br>2. ☐ Sometimes<br>3. ☐ Never
Q4.3.- Do you wash your hands with soap after toilet?	1. ☐ Always<br>2. ☐ Sometimes<br>3. ☐ Never
Q4.4.- Do you walk bare foot?	1. ☐ Always<br>2. ☐ Sometimes<br>3. ☐ Never
Q4.5.- Do you treat tap water before drinking?	1. ☐ Always<br>2. ☐ Sometimes<br>3. ☐ Never
Q4.6.- Do you defecate in open places?	1. ☐ Always<br>2. ☐ Sometimes<br>3. ☐ Never
Q4.7.- Do you wash fruits and vegetable before eating?	1. ☐ Always<br>2. ☐ Sometimes<br>3. ☐ Never
**Practices related to soil-transmitted helminth treatment attitude**	
Q4.8.- Have you ever heard about any treatment for human intestinal worm (deworming)	1. ☐ yes<br>2. ☐ No<br>Comment your answer…………………………………………………
Q4.9- Do you know a name of the drug used for the treatment of human intestinal worm?	………………………………………………………………………
Q4.10.- Do you know any traditional remedy for the treatment of human intestinal worm?	1. ☐ yes<br>2. ☐ No<br>Name it/them: ……………………………………………………

Thank you for your participation and for answering these questions.

The research team

Interviewer initials |__|__|__|

Supervisor Initials: |__|__|__|

Date: |__|__| |__|__|__|

Initial Data Entry |__|__|__|
