# Supplementary material for: Knowledge, attitudes, and practices related to soil-transmitted helminth infections among residents of Bata district, Equatorial Guinea; a cross-sectional study
Source: BMC Public Health. 2024 Jul 23;24:1962. doi: 10.1186/s12889-024-19528-0 (PMC11264363; doi:10.1186/s12889-024-19528-0)
Supplement: Supplementary file 3 — Supplementary Material 3: Supplementary material S2. Simplify version of the study main questionnaire for children aged 10 to 17 years [file 12889_2024_19528_MOESM3_ESM.pdf]

PARTICIPANT DATA COLLECTION FORM

" MINOR (10-17) YEARS OLD".

PROTOCOL: [*V1.0 Dec./06/20*]

[CONTROL OF *SOIL TRANSMITTED HELMINTHS IN EQUATORIAL GUINEA*]

Date of the survey:

Date		Month			Year		

Q1. SOCIO-DEMOGRAPHIC & ECONOMICAL DATA

Q1.1.-Sex
1. ☐ Male
2. ☐ Female

Q1.2.-Age
Q 1.2. 1.- Date of birth |__|__| |__|__|__| |__|__|__|__|
Date Month year

Q 1.2.2.- How old are you? |__|__| years

Q1.3.-What is the name of your community? ……………………………………………………………………………..

Q1.4.-How long has you been living here? ………………………………………………………………………………….

Q1.5.- Do you attend class?
1. ☐ Yes
2. ☐ No

Q1.5.1.- Which is your education level? ………………………………………………………………………………………….

Q1. 6. Which is your parents' principal occupation?
1. ☐ Farmer/ Fishermen
2. ☐ Civil servants
3. ☐ Trader
4. ☐ Unemployed

Q1.7 Living conditions

Q1.7.1 - In which type of house are you living?
1. ☐ Cement
2. ☐ Plank
3. ☐ Other……………………………………………………

Q1. 7.12.- How is the floor of your house?
1. ☐ Cemented
2. ☐ un-cemented

Q1.8. Sanitation

Q1.8.1- Do you use toilet
1. ☐ yes => skip question Q1.8.4
2. ☐ No => go to question Q1.8.4

Q1.8.2.- What type of toilet do you use?
1. ☐ Private/Modern
2. ☐ Communal latrine
3. ☐ Pit latrine

Q1.8.3 Type of toilet floor
1. ☐ Cemented
2. ☐ un-cemented

Q1.8.4 if you don't use toilet, could you specify defecation place
1. ☐ Behind house
2. ☐ Open places
3. ☐ Bush
4. ☐ Others ……………………………………………………………………………….

Q1.9.- Which is principal Domestic water sources?
1. ☐ Tap inside house
2. ☐ Tap out site house
3. ☐ Well
4. ☐ Stream
5. ☐ Other…….

Q1.10.- Is it possible to have indirect contamination of your Domestic water?
1. ☐ yes
2. ☐ No

Q1.11.- Do you have water Sources contaminated with feaces close to the house?
1. ☐ yes
2. ☐ No

Enviromental Sanitation

Q1.12.- Do you have sewage System connected to your house?
1. ☐ yes
2. ☐ No

Q.1.12.1.- If No, where do you drain the waste water? (toilet, kitchen)
1. ☐ River
2. ☐ Behind house
3. ☐ Underground
4. ☐ Others……………………………………………………………………………………………………….

Q2.- ASSESSMENT OF VOLUNTEER KNOWLEDGE TOWARDS THE DISEASE

Q2.1.- Have you ever heard about human intestinal worms?

1. ☐ yes
2. ☐ No

Q2.2.- Consider intestinal worm as a disease?

1. ☐ yes
2. ☐ No

Q2. 3.-Where did you hear about intestinal parasites (worms) for the first time?

1. ☐ At school
2. ☐ At home
3. ☐ At the health center/Hospital
4. ☐ During health campaign
5. ☐ Not answer

Q2.4- What do you think are the causes of human intestinal worms can you mention some principal causes?

1. ☐ ____
2. ☐ ____
3. ☐ ____
4. ☐ ____

Q2.5.- Do you know the local name of SHT infection

1. ☐ yes
2. ☐ No

Q2.6.- Which are the symptoms that show you that some has Human intestinal Worms

1. ☐ ____
2. ☐ ____
3. ☐ ____

Q2.7.- Do you know how any can contact STH

1. ☐ ____
2. ☐ ____
3. ☐ ____

Q2. 6.- Which of the following Symptoms are related with the Human Intestinal worms?

1. ☐ Abdominal pain
2. ☐ Anemia
3. ☐ Under weight- and malnutrition
4. ☐ Lack of concentration at school
5. ☐ Stunted growth
6. ☐ Lack of appetite
7. ☐ I don't know
8. ☐ Others ____

Q2. 7.- How can we prevent human Intestinal worm's?

1. ☐ ____
2. ☐ ____
3. ☐ ____
4. ☐ ____

Q3.- ASSESSMENT OF VOLUNTEER ATTITUDE TOWARDS THE DISEASE.

Q3.1.- According to you who can mainly suffer the disease?

1. ☐ Anyone
2. ☐ Children
3. ☐ Women
4. ☐ The Poor
5. ☐ I don't know
6. ☐ Others____

Q3.2.- If someone has Intestinal worms, where would you go for solution?

1. ☐ To the Hospital
2. ☐ To the Pharmacy (Auto- medication)
3. ☐ I will use home remedies
4. ☐ I can't tell
5. ☐ Others-____

Q.3.3.- Which treatment do you consider as better, Pharmaceuticals drugs or home remedies

1. ☐ Pharmaceuticals drugs
2. ☐ Both
3. ☐ Home remedies
4. ☐ I can't tell
5. ☐ I don't know

Q4.- ASSESSMENT OF VOLUNTEER PRACTICES TOWARDS THE DISEASE.

Q4.1.- Do you Wash your hands with soap before Eating?

1. ☐ Always
2. ☐ Sometimes
3. ☐ Never

Q4.2.- Do you Wash your hands with soap after toilet?

1. ☐ Always
2. ☐ Sometimes
3. ☐ Never

Q4.3.- Do you normally Walk barefoot?

1. ☐ Always
2. ☐ Sometimes
3. ☐ Never

Q4.4.- Do you process the tap water before drinking

1. ☐ Always
2. ☐ Sometimes
3. ☐ Never

Q4.5.- Do you defecated in open places?

1. ☐ Always
2. ☐ Sometimes
3. ☐Never

Q4.6.- Do you wash fruits and vegetable before Eating?

1. ☐ Always
2. ☐ Sometimes
3. ☐ Never

Q4.7.- Have you ever Heard about any treatment for human intestinal worm (deworming)

1. ☐ Yes
2. ☐ No

Please can you comment your answer……………………………………………………………………………………………………

Q4 7.1 Which is the name of the drug used? ……………………………………………………………………………………..

Q4. 8.- Do you know any Home remedy for STH

1. ☐ yes
2. ☐ No

Please name it…….

****

Thank you for participating and answering this questioner

The Research team

Interviewer initials: |__|__|__|

Supervisor Initials: |__|__|__|

Date: |__|__| |__|__|__| |__|__|__|_| Initial Data Entry: |__|__|__|
